# Is Intra-Aortic Balloon Pump Counterpulsation Sufficient to Treat Patients in Cardiogenic Shock, Undergoing Primary Percutaneous Coronary Intervention

**DOI:** 10.14740/cr415w

**Published:** 2015-12-16

**Authors:** Maria Paton, Lisa Ashton, Ian Pearson, Mohan Sivananthan

**Affiliations:** a University of Leeds, Leeds, UK; b Leeds Heart Centre, Leeds, UK

**Keywords:** Intra-aortic balloon pump, Cardiogenic shock, Primary percutaneous coronary intervention, Risk model

## Abstract

**Background:**

A high number of patients do not survive primary percutaneous coronary intervention (PCI) complicated by cardiogenic shock (CS), even when assisted with intra-aortic balloon pump (IABP) counterpulsation. There is no accepted consensus on who may most benefit from IABP counterpulsation, although previous retrospective studies have reported predictors of survival for patients undergoing PCI and cardiac surgery. To date, a risk model for emergency primary PCI patients has not been ascertained. The objective of this study was to identify independent predictors for in-hospital survival, to create a standardized risk model to predict patients who may require IABP insertion during primary PCI.

**Method:**

Retrospective data were from 165 patients who had undergone primary PCI with IABP due to CS complicating acute myocardial infarction (AMI), from September 2007 to 2010, and underwent logistic regression analysis, to evaluate the incremental risk factors associated with survival.

**Results:**

The overall in-hospital mortality was 32.1% (53 patients). The incremental independent predictors for in-hospital survival were: patient age of less than 60 years (OR: 0.303, 95% CI: 0.11 - 0.83, P < 0.02) and the use of IABP support alone, as opposed to in adjunction with inotropic support (OR: 3.177, 95% CI: 1.159 - 8.708, P < 0.025).

**Conclusion:**

This study illustrated an age of less than 60 years, and the use of IABP alone, to be independent predictors of in-hospital survival in patients with CS complicating AMI who undergo primary PCI assisted by IABP. No specific risk model could be determined.

## Introduction

Intra-aortic balloon counterpulsation (IABP), introduced clinically in 1968 [1], to support patients undergoing surgical revascularization, aims to augment coronary blood flow to increase myocardial oxygen supply, without an increase in myocardial workload [2, 3]. This is achieved through inflation during diastole (increasing coronary blood flow), and deflation during systole (reducing cardiac workload secondary to a reduced afterload) [4, 5].

In recent years, technological advances have led to an increase in IABP use in adjunct to percutaneous coronary intervention (PCI) [6-8]. Consequently, IABP insertion is now regarded as a class one recommendation in the management of acute myocardial infarction (AMI) in current European Society of Cardiology and American Heart Association guidelines [9].

AMI is associated with cardiogenic shock (CS) in 5-10% of patients [5, 10] with a high mortality rate of 50% [10, 11]. IABP has been shown to improve prognosis in these cases [12], therefore is an important factor in improving survival rates. Additional fundamental confounders may also influence the efficiency of IABP (patient age, timing of insertion, and intervention undertaken); however, evidence for the impact of these is contradictory [3, 13].

The European System for Cardiac Operative Risk Evaluation [14] is an objective method of risk stratification that aims to consider such factors to predict early mortality in IABP patients post-cardiac surgery [14, 15]. Although utilized by many surgeons as an indicator of appropriate IABP insertion, it underestimates mortality rates in patients inserted with an IABP peri- and post-operatively [16] and therefore cannot be successfully applied in primary PCI procedures.

A three-fold difference in the use of IABP between centers demonstrates this variation in indication parameters [17]. Lack of consensus is particularly significant considering the 47% increase in IABP utilization over the last few years [17]. This may be attributed to a deficiency in the evidence base, with a lack of randomized study design due to its introduction prior to the Medical Device Amendment in 1976 [18].

Further investigation is necessary to establish a model and hence specific predictors that will identify whether high-risk patients will benefit from an assistance device during PCI [19]. The primary aim of the present investigation was to develop a risk stratification model, to provide insight into which primary PCI patients IABP use is most beneficial, as well as the factors which have the highest predictive power of patient outcome.

## Method

### Patient population

The study population initially consisted of 175 patients who were recruited retrospectively from a single tertiary hospital. All patients underwent emergent primary PCI as a standard treatment of AMI between September 2007 and September 2010, and received IABP in response to suspected CS (defined as the presence of low systolic blood pressure (< 90 mm Hg) resulting in cardiac insufficiency with additional signs of hypoperfusion unaltered by fluid resuscitation) [20].

The initial population of 175 patients were reduced to 165 with 10 patients being excluded due to involvement in an alternate, randomized IABP study and a further.

### IABP

All patients received a Datascope 8Fr sheathless balloon catheter (Sheathless balloon catheter, Datascope Co. Ltd, Cambridgeshire, UK) and introducer which were inserted percutaneously via the femoral artery and connected to a Datascope computerized portable console (CS100 IABP, Datascope Co. Ltd, Cambridgeshire, UK). An experienced operator (consultant cardiologist) performed IABP insertion in the catheterisation laboratory under radiological control. Correct position of the catheter was evaluated by fluoroscopy in all cases. IABP was programmed to a 1:1 inflation ratio with full balloon augmentation, triggered by ECG. The IABP was left *in situ* post-procedure, with the decision to remove the IABP left to the discretion of the operator.

### Statistical analysis

Data were extracted from the electronic departmental cardiac database (Cardiobase 6.0, Magnus Medical Software, UK). Patient characteristics, including age, sex, and co-morbidities were documented as well as MI location, initial systolic blood pressure, initial diastolic blood pressure, operator, additional circulatory support, procedural complications, and vital status at hospital discharge. All data were entered into a commercially available spreadsheet (Excel 2007, Microsoft Corp., Washington, USA) and subsequently transferred to PASW statistics software (version 18) for analysis. The patients were then subdivided into two groups depending on survival (group 1) or non-survival (group 2) state at discharge.

### Compare mortality

Chi-square statistical analysis was utilized to compare age, sex, initial systolic BP, and the presence of ST elevation, renal disease, left main stem disease, triple vessel disease and single disease variables between group 1 and group 2.

A multivariate logistic regression analysis was used to determine independent predictors of in-hospital survival. A full model including all selected variables was primarily obtained, followed by a stepwise model generated by including variables identified as statistically significant (P < 0.05). The method of fit of the model was measured by the reduction in log-likelihood ratio, Chi-square statistic.

## Results

### Demographic analysis

The baseline characteristics of the cohort are shown in Table 1. An overall in-hospital mortality rate of 32.1% (53 patients) was demonstrated. Seventy-one (64.3%) and 44 (83.0%) in the survivor and non-survivor groups respectively were aged 60 or over (median: 69 years), signifying a single significant difference (P < 0.014) between the two subgroups. Further demographic analysis demonstrated no significant difference in relation to multiple vessel disease (P > 0.653), single vessel disease (P > 0.925), ST-elevation presence on initial ECG (P > 0.132), and sex (P > 0.721). Initial systolic BP demonstrated a trend towards significance (P > 0.076).

**Table 1:** Results of X^2^ Analysis of the Demographic Data

Survival in hospital	Number of patients	Age 60+ (%)	Female (%)	Initial systolic BP below 90 mm Hg (%)	ST elevation on initial ECG (%)	Left main stem disease (%)	Multiple vessel disease (≥ 2)	Single vessel disease (%)
Survivors	112	64.3%	31.3%)	69.6%	78.6%	17.0%	36.6%	63.4%
Non-survivors	53	83.0%	28.3% (15/53)	50.9%	86.8%	24.5%	35.8%	64.2%
X^2^		6.047	0.653	3.254	2.266	1.1317	0.204	0.009
P value		0.014	0.721	0.076	0.132	0.251	0.652	0.925

### Multivariate logistic regression

To allow for variable interactions, multivariate logistic regression analysis was carried out in a forward stepwise elimination model to determine independent predictors of in-hospital survival. The variables entered into the model included: procedural complications, location of disease (vessels), initial diastolic and systolic BP, circulatory support, sex and age.

The regression analysis (Table 2) demonstrated a patient age of less than 60 years (OR: 0.303, 95% CI: 0.11 - 0.83, P < 0.02) and the use of IABP support alone (OR: 3.177, 95% CI: 1.159 - 8.708, P < 0.025), compared to in adjunction with inotropic support, were statistically significant predictors of in-hospital survival. All other variables were not deemed significant and therefore not included in the stepwise model.

**Table 2:** General Logistical Regression Results

Model	Variables in the Equation	B	SE	Log likelihood	Degrees of freedom	X^2^ significance statistic	Odds ratio	Lower 95% CI	Upper 95% CI
0				140.606	1	0	0.443		
1	Age (1) (60-)	-1.191	0.504	134.203	1	0.018	0.304	0.113	0.815
2	Age (1)	-0.005	0.515			0.02	0.303	0.11	0.83
	Support (1)	1.156	0.514	129.138	2	0.025	3.177	1.159	8.708

**Figure 1:**
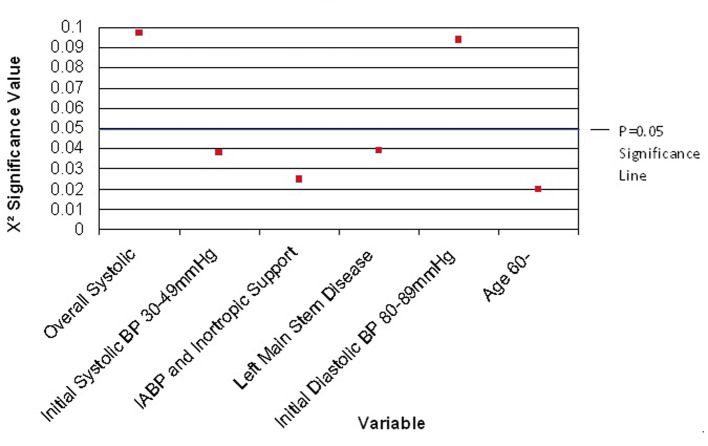
Variables with a trend towards X^2^ significance.

Particular sub-categories of variables showed significance (Fig. 1). These included an initial systolic BP range of 30 - 49 mm Hg (P < 0.038), and left main stem disease (P < 0.039); however, overall initial systolic BP and location of disease did not show significance and so were not deemed suitable for inclusion in the model.

**Figure 2:**
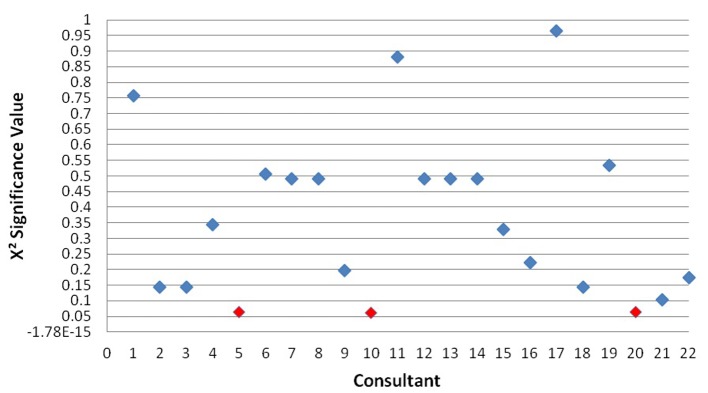
X^2^ significance of operators in predicting survival outcome.

A separate forward stepwise logistical regression indicated that overall operator variation was not shown to be an independent predictor of survival at discharge (P < 0.174). However, a number of exceptions were highlighted with a trend towards significance: operator 5 (P < 0.063), operator 10 (P < 0.061), and operator 20 (P < 0.063) (Fig. 2).

## Discussion

### Overall mortality rate

The overall in-hospital mortality rate in this study for patients undergoing primary PCI with CS, inserted with an IABP was found to be 32.1%. This is comparable to other retrospective observational studies investigating patients requiring an IABP due to CS [7, 21, 22]. A possible explanation for the observed mortality benefit could be improved myocardial perfusion, which itself has been shown to be an independent predictor of in-hospital survival [23, 24]. However, recent investigation into the effects of IABP on coronary pressure in patients with stenoses has concluded that diastolic coronary perfusion distal to the coronary obstruction is not augmented [12]; therefore further investigation into the perfusion benefit of IABP is required.

The high percentage of in-hospital mortality, which is clinically significant, indicates IABP is insufficient in some patients, and also an inefficient use of the IABP. Therefore, it is necessary to further investigate the independent predictors of survival surrounding its use, to improve utilization.

### Risk stratification model

The primary findings from this study are firstly, age and the absence of inotropic agents are independent predictors of in-hospital survival after primary PCI in adjunction to IABP. Secondly, initial systolic BP of 30 - 49 mm Hg and the presence of left main stem disease may influence outcomes, although these are not deemed statistically significant in this study. Whilst predictive factors were identified, no comprehensive model can be designed from these data to identify those patients most at risk, and therefore most likely to benefit from IABP insertion.

Previous risk stratification models from patients undergoing CABG [21] have demonstrated models can be achieved; however, these cannot be applied to this patient population due to the nature of the clinical data and the diverse procedural indications. This may indicate that separate models are necessary for each intervention, potentially overcomplicating guidelines and restricting use within the acute clinical setting.

However, the present study is consistent with findings from the widely validated Mayo Clinic risk prediction model [22], where age was a significant factor in predicting survival, and the presence of left main stem disease demonstrated a similar trend. Independent predictors in the Mayo Clinic model were specifically incorporated in the present studies patient demographics. Whilst this restricted a more generalized independent assessment of the patient criteria, limiting comparable analysis, the current study could be considered a more specific sub-analysis of the Mayo Clinic model.

Therefore, it is interesting that there are differences in the degree of significance of vessel disease; this may be attributed to the largely differing sample size. Although comparable to similar studies [23, 25], a larger sample size may have allowed for detection of significant variables more readily. This characteristically small sample size may demonstrate the general underutilization of IABP counterpulsation.

Additionally terminology is inconsistent between studies investigating IABP. Collated data are frequently based on “high-risk” patients only [19, 26, 27], the definition of which is not standardized. Previously, patients have been classified by the presence of multi-vessel disease, left main stem disease, severity of left ventricular dysfunction, age, or increased right heart pressures [27, 28]. As a result, comparison of these studies is difficult, and in many cases unjustified.

Furthermore, follow-up time periods differ greatly within studies and have been reportedly gained at up to 5 years post-procedure [26]. The duration of follow-up in the present study was limited to the in-hospital phase, and therefore long-term outcome could not be reported. However, IABP is merely considered a bridging therapy [29] and therefore long-term survival is determined by many other factors which must be taken into consideration when making a decision on implantation.

### Age and severity of vessel disease

Age was the largest independent predictor of survival in this study. In accordance with a previous report, we found that survival is higher in younger patients with CS treated with IABP [5]. This is comparable to the prospective randomized SHOCK (Should We Emergently Revascularise Occluded Coronaries for Cardiogenic Shock) registry [30].

A significant link has been proved between patient age and specifically the number and severity of coronary lesions observed, as well as an increase in co-morbidities [31]. The increased need for IABP in recent years [7, 8, 17] has been credited predominantly to the increased age and disease severity, resulting in increased high risk PCI procedures. This has implications in our data where no age limit was defined for undertaking such a procedure.

Whilst the presence of multi-vessel disease was not significantly different between survivors and non-survivors, the existence of left main stem disease did show a trend towards significance in predicting in-hospital outcome. Therefore this study reiterates the relationship noted between age and the severity of lesions observed.

### Additional inotropic support

Whilst the use of IABP with inotropes has been associated with increased survival [14], this study showed IABP support alone was associated with a favorable outcome at discharge. However, inotropic support is often only undertaken in patients at a perceived higher risk adverse events, and therefore lower survival prospects. Inotropic support also leads to increased peripheral vasodilation and further hypotension in some patients [32]; counteracting the effect of IABP counterpulsation, this association would require further elucidation.

### Systolic BP between 30 and 49 mm Hg

Although an initial systolic BP of 30 - 49 mmHg showed a trend towards significance, specific time periods for BP measurement and IABP initiation were not recorded and therefore, not analyzed.

Prompt revascularization is considered the ultimate goal for treating patients with AMI complicated by CS [6]. Currently, ACC/AHA guidelines for PCI recommend that IABP support should be retained for patients at severe hemodynamic compromise [9, 33]. In the absence of a definitive model to guide utilization of IABP, operators will continue to treat lesions first whilst monitoring hemodynamic stability. However, it has been demonstrated that in-hospital survival for early revascularization and medical therapy alone is not significantly different [30], which was attributed to increased use of IABP in the patients undertaking medical therapy [12, 34].

As the present study involves only patients treated with IABP therapy, the influence of IABP independently on outcome cannot be predicted. However, patients with an initial BP of 30 - 49 mm Hg were likely to have had IABP insertion prior to procedure, and therefore it can be assumed that earlier insertion increases in-hospital survival. This is reiterated in current AHA/ACC guidelines [28], stating that IABP insertion just before coronary instrumentation results in increased patient outcomes in those with at least borderline hemodynamic instability.

It continues to remain unclear in current literature which specific patients would benefit from insertion of IABP prior to procedure and therefore these data are timely [31, 35]. This study goes part way in addressing this, as timing of IABP therapy initiation should be related to the hemodynamic stability of the patient.

### Overall mortality rate

The overall in-hospital mortality rate in this study for patients undergoing primary PCI with CS, inserted with an IABP was found to be 32.1%, with a 1-year mortality of 26.8%. This is comparable to other retrospective observational studies investigating patients requiring an IABP due to CS [7, 25, 26]. A possible explanation for the observed mortality benefit could be improved myocardial perfusion, which itself has been shown to be an independent predictor of in-hospital survival [23, 24]. However, recent investigation into the effects of IABP on coronary pressure in patients with stenoses has concluded that diastolic coronary perfusion distal to the coronary obstruction is not augmented [12]; therefore further investigation into the perfusion benefit of IABP is required.

### Operator bias

It was determined from the present study that the specific operator had no significant impact on patient survival to discharge. However, many other studies discuss operator bias as a limitation without statistically analyzing its impact on their results [25, 26, 36]. In fact, no other studies encompassing the direct analysis of operator variation have been found, and therefore this limitation is based merely on speculation. The results presented here indicate some operators show trends towards significance and therefore operator impact may require further study.

### Conclusion

IABP counterpulsation in patients undergoing primary PCI for AMI complicated by CS is beneficial in patients below 60 years of age, with no adjunctive inotropic treatment. Whilst a model was not derived from these data, it is still a worthwhile goal in order to provide appropriate treatment earlier. It may also be useful to investigate alternative modes of blood pressure augmentation for the more acutely ill patient.

Small, retrospective studies cannot eliminate bias and confounding factors, therefore the evidence solely provided by such studies is insufficient to endorse new guidelines. That set aside, these data are of value, and highlight the need for further more detailed exploration.
